# Conditions Conducive to the Glutathionylation of Complex I Subunit NDUFS1 Augment ROS Production following the Oxidation of Ubiquinone Linked Substrates, Glycerol-3-Phosphate and Proline

**DOI:** 10.3390/antiox11102043

**Published:** 2022-10-17

**Authors:** Kevin Wang, Jonathan Hirschenson, Amanda Moore, Ryan J. Mailloux

**Affiliations:** The School of Human Nutrition, Faculty of Agricultural and Environmental Sciences, McGill University, Sainte-Anne-de-Bellevue, Montreal, QC H9X 3V9, Canada

**Keywords:** mitochondria, glutathionylation, complex I, hydrogen peroxide, glycerol-3-phosphate dehydrogenase, proline dehydrogenase

## Abstract

Mitochondrial complex I can produce large quantities of reactive oxygen species (ROS) by reverse electron transfer (RET) from the ubiquinone (UQ) pool. Glutathionylation of complex I does induce increased mitochondrial superoxide/hydrogen peroxide (O_2_^●−^/H_2_O_2_) production, but the source of this ROS has not been identified. Here, we interrogated the glutathionylation of complex I subunit NDUFS1 and examined if its modification can result in increased ROS production during RET from the UQ pool. We also assessed glycerol-3-phosphate dehydrogenase (GPD) and proline dehydrogenase (PRODH) glutathionylation since both flavoproteins have measurable rates for ROS production as well. Induction of glutathionylation with disulfiram induced a significant increase in O_2_^●−^/H_2_O_2_ production during glycerol-3-phosphate (G3P) and proline (Pro) oxidation. Treatment of mitochondria with inhibitors for complex I (rotenone and S1QEL), complex III (myxothiazol and S3QEL), glycerol-3-phosphate dehydrogenase (iGP), and proline dehydrogenase (TFA) confirmed that the sites for this increase were complexes I and III, respectively. Treatment of liver mitochondria with disulfiram (50–1000 nM) did not induce GPD or PRODH glutathionylation, nor did it affect their activities, even though disulfiram dose-dependently increased the total number of protein glutathione mixed disulfides (PSSG). Immunocapture of complex I showed disulfiram incubations resulted in the modification of NDUFS1 subunit in complex I. Glutathionylation could be reversed by reducing agents, restoring the deglutathionylated state of NDUFS1 and the activity of the complex. Reduction of glutathionyl moieties in complex I also significantly decreased ROS production by RET from GPD and PRODH. Overall, these findings demonstrate that the modification of NDUFS1 can result in increased ROS production during RET from the UQ pool, which has implications for understanding the relationship between mitochondrial glutathionylation reactions and induction of oxidative distress in several pathologies

## 1. Introduction

Protein S-glutathionylation is a cellular redox signaling pathway that involves the reversible covalent modification of proteinaceous cysteine residues [1]. Glutathionylation occurs in response to oxidation of the reduced glutathione (GSH) pool, which drives activation/deactivation of proteins to elicit rapid cell responses to intra- and extracellular stimuli (reviewed in [1,2,3]). Restoration of the reducing potential of the glutathione pool by NADPH and glutathione reductase induces deglutathionylation. Mammalian glutaredoxins (GRX1; cytoplasm and intermembrane space, GRX2; mitochondrial matrix), small heat stable proteins that are part of the thioredoxin superfamily, catalyze reversible glutathionylation in response to GSH pool oxidation and reduction, respectively [4]. Glutathione S-transferase (GST) isoform P also catalyzes these reactions, utilizing GSH to modify proteins in several cellular compartments, which demonstrates that glutathionylation events do not exclusively occur when GSH is oxidized by H_2_O_2_ [3].

**Figure 1:**
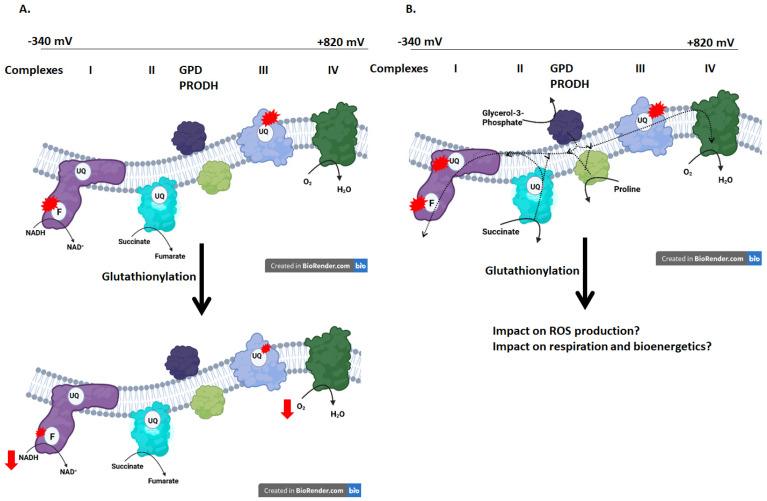
(**A**) Glutathionylation inhibits the NADH oxidizing and ROS generating activity of complex I. Modification can occur on many complex I subunits but the addition of glutathione to these components has been omitted for clarity. Modification of NDUFS1, which forms part of the nicotinamide binding site in complex I, disables ROS production by complex I and ROS sites upstream from complex I by inhibiting electron transfer from NADH to FMN (I_F_) and the UQ binding site (I_UQ_). Other UQ binding sites are denoted in the other complexes. Note the UQ site in complex III (III_UQ_) is a ROS source as well. (**B**) Reverse electron transfer (RET) from nutrient oxidizing enzymes that reduce the UQ pool (complex II or succinate dehydrogenase, GPD; glycerol-3-phosphate dehydrogenase, PRODH; proline dehydrogenase) can stimulate ROS production by complex I. The effect of glutathionylation on RET-induced ROS production by complex I remains under studied. SPECIAL NOTE: Mitochondria can contain up to 16 ROS generators, 12 of which are associated with bioenergetics and oxidative phosphorylation. These other sites have been excluded for clarity. Figures were generated using Biorender.com.

Glutathionylation reactions are ubiquitous with ~883 and >2000 proteins reported to be modified in liver and contracting muscle tissue, respectively, as well as in many other mammalian cells [5,6,7,8]. At the cellular level, reversible glutathionylation of proteins is required to regulate a myriad of functions ranging from chemotaxis and neutrophil extracellular trap formation to ion transport, gene expression, and metabolism [9]. In mitochondria, the glutathionylation of proteins in response to a decrease in the GSH/GSSG ratio inhibits energy metabolism, solute transport, apoptosis, and ATP production, while promoting mitochondrial fusion and protein folding [10]. Complex I was the first glutathionylation and GRX2 target discovered in mitochondria [11]. Modification of complex I subunit NDUFS1 prevents NADH metabolism but also turns down ROS production when Krebs cycle linked nutrients are being oxidized (Figure 1A) [11,12,13]. NDUFS1 is required for complex I activity and forms part of the NADH binding and oxidation site in theN-module of the enzyme. Flavin mononucleotide (FMN), which is found next to the active site, is required to facilitate electron flow from NADH to ubiquinone (UQ) in complex I (Figure 1A). The FMN moiety is also an important ROS source in complex I because it cycles between oxidized and semi-reduced states during electron transfer from NADH to UQ [14]. Glutathionylation reportedly prevents ROS production through NDUFS1 modification by blocking the nicotinamide binding site, which limits the reduction of FMN during NADH oxidation (Figure 1A) [11,13].

Complex I can have a high rate for ROS production during reverse electron transfer (RET) from the UQ pool as well [15]. This occurs through oxidation of nutrients that by-pass NADH generating pathways in the Krebs cycle and donate electrons directly to the UQ pool (Figure 1B). Complex II is the canonical stimulator of RET-induced ROS production but other flavoproteins that couple nutrient oxidation to the direct reduction of the UQ pool, like GPD and PRODH, can also induce high rates of ROS production by complex I (Figure 1B). RET-induced ROS production by complex I has been linked to several pathologies such as heart disease and the progression of cataracts [16,17]. Additionally, RET-induced disease progression can be blocked by complex I inhibitors that compete for UQ binding site like rotenone, mitochondria-targeted S-nitrosylation agents, and S1QELS [18,19,20]. As noted above, several studies have demonstrated glutathionylation inhibits ROS production during NADH oxidation. However, whether this is the case following RET from the UQ pool has remained unexplored. Indeed, Taylor et al. showed glutathionylation does increase ROS genesis by complex I [21] and it was hypothesized later this may be due to the oxidation of UQ-linked nutrients [22]. Several studies also showed glutathionylation of complex I in pathological states such as heart disease and cataract development is associated with higher mitochondrial ROS production and oxidative distress [14,23]. Additionally, reversal of glutathionylation with reductants like DTT restores complex I activity and mitochondrial ATP production and overexpression of GRX2 protects from heart disease by promoting complex I deglutathionylation and preventing oxidative damage after exposure of the myocardium to doxorubicin [23,24]. It was also recently shown chemical glutathionylation catalysts augmented succinate, glycerol-3-phosphate and proline-mediated production of ROS by the respiratory chain [25]. Taken together, metabolism of UQ-linked nutrients could account for the increased ROS production observed in pathological states. This may be due to the glutathionylation of NDUFS1 and electron accumulation in complex I after RET.

In the present study, we investigated G3P and Pro fueled ROS production during RET after inducing glutathionylation. These studies were conducted in tandem with assessments of the glutathionylation status of NDUFS1. We also interrogated (1) the glutathionylation state of GPD and PRODH using BioGEE switch assays and (2) the effect of glutathionylation on GPD and PRODH activity and its impact on forward electron transfer (FET) reactions in the electron transport chain. We discovered here that modification of the NDUFS1 subunit with glutathione and inhibition of complex I accounted for the increase in ROS genesis during the oxidation of UQ-linked substrates. Furthermore, we show GPD and PRODH are not glutathionylation targets and that oxidation of UQ-linked substrates under our glutathionylation conditions also augments ROS production by complex III. The importance of these fundamental findings and their implications in understanding the relationships between ROS and glutathionylation of complex I in the contexts of physiology and disease are discussed herein.

## 2. Experimental

### 2.1. Chemicals

S1QEL, S3QEL, iGP, TFA, rotenone, myxothiazol, succinate, glycerol-3-phosphate, proline, disulfiram, Triton X-100, D-mannitol, Hepes, sucrose, ethylene glycol-bis (β-aminoethyl ether)-N,N,N′,N′-tetraacetic acid (EGTA), horseradish peroxidase (HRP), superoxide dismutase, defatted BSA, antimycin A, rotenone, reduced glutathione, DMSO, NAD^+^, Bradford reagent, and 2,6-dichlorophenolindophenol (DCPIP) were purchased from Sigma-Aldrich. Biotinylated glutathione ethylester (BioGEE), chemiluminescent substrate, streptavidin beads (magnetized Dynabeads, ThermoFisher, Waltham, MA, USA), and Amplex UltraRed (AUR) reagents were supplied by Thermofisher. Complex I immunocapture kit, anti-PSSG, anti-GPD, anti-PRODH, anti-NDUFS1, and goat anti-mouse and rabbit HRP conjugate were supplied by Abcam (Cambridge, UK).

### 2.2. Isolation of Liver Mitochondria

Animals were cared for in accordance with the principles and guidelines of the Canadian Council on Animal Care and the Institute of Laboratory Animal Resources (National Research Council). All procedures using mice were approved by the Facilities Animal Care Committee (FACC) in the Faculty of Agricultural and Environmental Sciences at McGill University. Male C57BL6N mice were purchased at 9-weeks of age from Charles River Laboratories and were housed at 25 °C in the Small Animal Care Unit on a 12 h day/night light cycle (lights on at 07:00 h) and provided free access to water and a standard chow. Mice were euthanized by cervical dislocation under heavy anesthesia (5% isoflurane) at approximately 10-weeks of age and livers were collected and placed in an ice-cold MESH buffer (mannitol (220 mM), EGTA (1 mM), sucrose (70 mM), and HEPES (10 mM), pH 7.4).

All steps for the isolation of mitochondria were performed on ice or at 4 °C. Livers were washed in MESH, cut into pieces, and washed again to remove excess blood. Liver pieces were minced on a Teflon watch glass and then placed in a homogenizing tube containing MESH supplemented with 0.5% (*w*/*v*) defatted BSA (MESH-B). Liver pieces were homogenized using the Potter-Elvejham method and a variable speed reversible homogenizer (Glas-Col). Homogenates were centrifuged at 800× *g* for 9 min to pellet cellular debris and nuclei. The supernatant was collected and centrifuged at 4000× *g* for 20 min. The supernatant was decanted, and the sides of the centrifuge tube were carefully cleaned with a Kimwipe to remove any residual fat. The pellet was resuspended in ~500 µL MESH to give a final concentration of protein equivalents to mitochondria of ~15–20 mg/mL. Protein equivalents to mitochondria were determined by a Bradford assay using BSA as a standard. Mitochondria were stored on ice until experimentation.

### 2.3. Interrogation of Mitochondrial O_2_^●−^/H_2_O_2_ Production

Samples were diluted to 0.5 mg/mL in the individual wells of a 96-well black microplate with clear bottoms containing MESH. Mitochondria were allowed to equilibrate for a few minutes. Samples were then treated with disulfiram (final concentration of 50–1000 nM) and 1 mM GSH. Reactions devoid of disulfiram served as the control. Following a 10 min incubation at 37 °C, mixtures were supplemented with horseradish peroxidase (3 U/mL final concentration) and superoxide dismutase (15 U/mL final concentration). Since SOD converts O_2_^●−^ to H_2_O_2_, rates of production are expressed as rate of H_2_O_2_ production (however, it is worthy to note that ROS sources in mitochondria generate a mixture of O_2_^●−^ to H_2_O_2._ Thus, although rates of production are expressed as described above, the capacity of ROS sources to produce both O_2_^●−^ to H_2_O_2_ is acknowledged throughout the text— e.g., O_2_^●−^/H_2_O_2_ production). Amplex UltraRed (AUR; 10 µM), and G3P or Pro were then added to each well to start the reactions. The final concentration of G3P and Pro was 1 mM. The conversion of AUR to its oxidized form in the presence of H_2_O_2_ was tracked at excitation and emission wavelengths 565 nm:600 nm and measured kinetically every 30 s for 5 min at 37 ℃ using a Cytation 5 Multimode Plate Reader (Biotek) equipped with Gen5 software. Results were normalized to protein equivalents to mitochondria and background fluorescence (mixtures containing all reaction components except substrates). Rates of production were calculated using AUR calibration curves that were developed using H_2_O_2_ as a standard.

### 2.4. Evaluation of O_2_^●−^/H_2_O_2_ Sources in Liver Mitochondria

To ascertain the source of ROS during the oxidation of G3P or Pro following disulfiram treatment, mitochondria were incubated in complex I inhibitors rotenone (1 µM) and S1QEL 1.1 (1 µM) and/or complex III inhibitors myxothiazol (4 µM; Q_P_ inhibitor) and S3QEL (1 µM). Atpenin A5 (10 µM), which blocks the UQ binding site in complex II, was also included in all experiments to prevent electron back flow through succinate dehydrogenase. Mitochondria were also preincubated in GPD and PRODH inhibitors, iGP (1 µM) or tetrahydro-2-furonic acid (TFA; 1 mM). Intact mitochondria were diluted to 0.5 mg/mL in reaction chambers containing MESH and then supplemented with rotenone, myxothiazol and/or rotenone with atpenin A5 and then incubated for 10 min at 37 °C. Samples were then supplemented with 1000 nM disulfiram and 1 mM GSH and incubated for an additional 10 min at 37 °C. Mitochondria were then treated with the AUR reagents listed above. Reactions were initiated by the addition G3P or Pro to a final concentration of 1 mM. The rate of H_2_O_2_ production was measured and calculated as described above.

**Figure 2:**
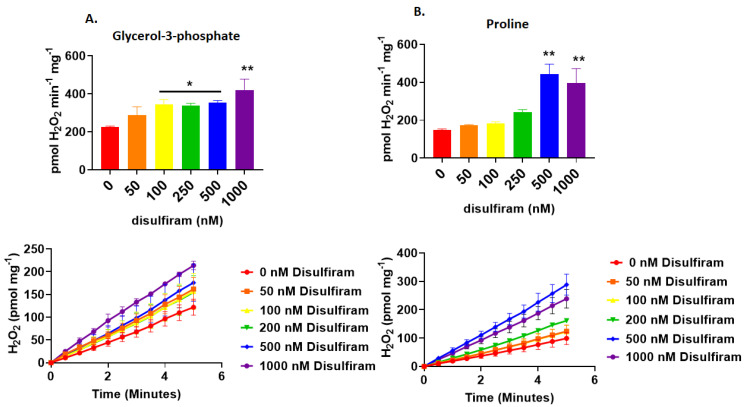
Chemical glutathionylation catalyst disulfiram augments O_2_^●-^/H_2_O_2_ production by mitochondria oxidizing (**A**) glycerol-3-phosphate and (**B**) proline. Estimates for the rate of production were determined using isolated mitochondria treated with different doses of disulfiram (0–1000 nM), 1 mM GSH, and AUR reagents. N = 4, mean ± SEM, 1-way ANOVA with a post-hoc Fisher’s LSD test. * *p* ≤ 0.05, ** *p* ≤ 0.01. Representative traces were also supplied for each determination.

### 2.5. GPD and PRODH Activity

Mitochondria were diluted to 5 mg/mL in MESH containing 0.01% (*v*/*v*) Triton X-100 (MESH-T) and incubated on ice for 20 min to permeabilize the inner membrane [26]. Mitochondrial permeability was assessed by measuring malate dehydrogenase activity as described in [26]. Samples were diluted to 0.5 mg/mL in the individual wells of a 96-well black microplate with clear bottoms containing MESH. Reactions were supplemented with rotenone (1 µM), myxothiazol (4 µM), and atpenin A5 (10 µM) and then treated with 2,4-dichloroindophenol (DCPIP; 25 µM), phenazine methosulfate (PMS, 10 µM), and 1 mM G3P or Pro. Control reactions included the addition of 10 µM iGP to inhibit GPD or 1 mM TFA for PRODH, respectively. Activities were calculated using the Beer-Lambert law (ε_610_ = 20.1 mM^−1^ cm^−1^) and normalized to background absorbance.

### 2.6. Measurement of PSSG Adducts

Samples were then diluted to 1 mg/mL in RIPA lysis buffer containing Laemmli tracking solution and 25 mM NEM and incubated at 100 °C for 10 min. 40 µg of protein sample was then electrophoresed through a 10% isocratic denaturing gel. Samples were transferred to nitrocellulose membranes and blocked for 1 h at room temperature under constant agitation using TBS-T containing 5% (*w*/*v*) non-fat skim milk. Membranes were washed and probed overnight at 4 °C using anti-PSSG antibody (protein glutathione mixed disulfide; 1/500). Membranes were then washed, incubated in secondary antibody-HRP conjugate, and bands visualized using chemiluminescent substrate (Thermofisher, Waltham, MA, USA) and a Licor C-Digit Scanner (Licor Biosciences, Lincoln, NE, USA). Samples electrophoresed with 2% (*v*/*v*) β-mercaptoethanol served as a control for PSSG anti-serum specificity.

### 2.7. BioGEE Switch Assays

For the BioGEE switch assays, livers were extracted, homogenized, and mitochondria isolated as described above using buffers supplemented with NEM. Mitochondria were diluted to 4 mg/mL in a RIPA lysis buffer and desalted using Zeba micro spin columns (ThermoFisher, Waltham, MA, USA). The eluant was collected and treated with 5 mM dithiothreitol (DTT) to reduce any protein-glutathione adducts. Samples were then desalted as described above and treated with biotinylated glutathione ethyl ester (BioGEE; 1 mM) and disulfiram (1 mM) and incubated for 30 min at 37 °C. For control experiments, BioGEE and disulfiram treated samples were also supplemented with 2% (*v*/*v*) β-mercaptoethanol to reduce BioGEE-modified proteins. Samples were then supplemented with streptavidin-modified magnetic beads (Dynabeads; Invitrogen, ThermoFisher, Waltham, MA, USA) and incubated overnight at 4 °C under constant agitation. Beads were magnetically separated from supernatant using a magnetic rack (BioRad, Hercules, CA, USA), washed twice with MESH, and then heated at 100 °C for 15 min to separate proteins from antibodies. The beads were removed using the magnetic rack and the resulting supernatant was treated with Laemmli buffer and electrophoresed and subjected to electroblotting as described above. Membranes were probed for GPD (anti-GPD; 1/1000) and PRODH (anti-PRODH; 1/1000) overnight, followed by a 1 h incubation in secondary HRP conjugate. Immunoreactive bands were visualized as described above.

### 2.8. Complex I Immunocapture

**Figure 3:**
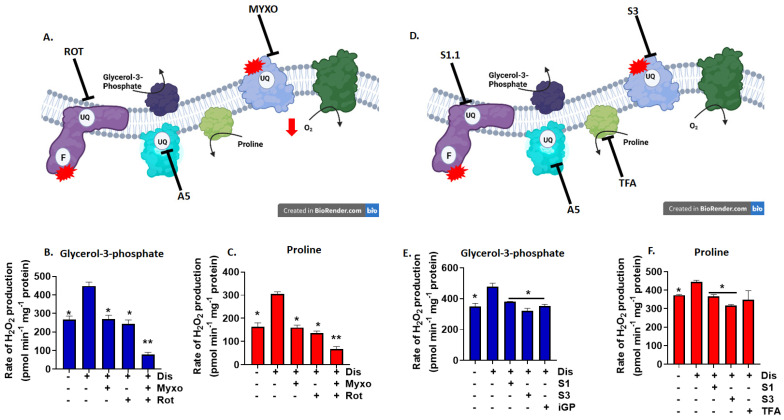
Complex I and III, GPD, and PRODH inhibitors impede the genesis of O_2_^●−^/H_2_O_2_ by mitochondria oxidizing G3P or Pro. (**A**) Diagram depicting the sites of action for rotenone (ROT, I_Q_ site; complex I) and myxothiazol (MYXO, III_Q_ site; complex III). Atpenin A5 (A5) is a II_Q_ inhibitor included to prevent reverse flow to complex II. Diagram was generated using Biorender.com. Mitochondria were fueled with G3P (**B**) and Pro (**C**), treated with 1000 nM disulfiram and 1 mM GSH and incubated with rotenone/myxothiazol/atpenin A5. (**D**) Diagram depicting the sites of action for S1QEL (S1, I_Q_ site; complex I), S3QEL (S3, III_Q_ site; complex III), iGP (GPD; glycerol-3-phosphate dehydrogenase), and tetrahydro furonic acid (TFA, PRODH; proline dehydrogenase). Diagram was generated using Biorender.com. Mitochondria were fueled with G3P (**E**) and Pro (**F**)**,** treated with 1000 nM disulfiram and 1 mM GSH and then incubated in S1, S3, or iGP (**E**) or TFA (**F**). Controls consisted of reactions devoid of disulfiram and respiratory inhibitors. N = 4, mean ± SEM, 1-way ANOVA with a post-hoc Fisher’s LSD test. * *p* ≤ 0.05, ** *p* ≤ 0.01.

Briefly, mitochondria were diluted to 2 mg/mL in MESH, treated with disulfiram (5000 and 10,000 nM) or diamide (1000 and 5000 µM) + 1 mM GSH, and incubated for 10 min at 37 °C. Complex I was the immunocaptured according to the manufacturer’s instructions (Abcam, Cambridge, UK). Samples were diluted in Laemmli buffer +/−2% (*v*/*v*) β-mercaptoethanol. The β-mercaptoethanol was included to reduce protein glutathione disulfide adducts (PSSG) and confirm the specificity of the PSSG anti-serum. Twenty microliters of sample was loaded in each well and samples were electrophoresed and transferred to nitrocellulose membranes as described above. Membranes were blocked and then probed with anti-NDUFS1 (1/1000) or anti-PSSG (1/1000) over night. Gel loading was conducted in a way to ensure NDUFS1 and PSSG could be probed with antibodies on the same membrane to allow for band alignment (Figure 3A,B). To do this, samples were loaded in duplicate and the membrane was cut vertically to probe for NDUFS1 and PSSG. Bands were visualized using chemiluminescent substrate and the Licor imager as described above.

### 2.9. Seahorse XFe24 Assays

Mitochondria were diluted to 0.5 mg/mL in MESH, and 50 μL of the diluted mitochondria was loaded into a Seahorse tissue culture (TC) plate well. The TC plate was centrifuged at 2000× *g* for 20 min at 4 °C to adhere the mitochondria to the bottom of the wells and then treated with 350 μL of respiration buffer (10 mM KH_2_PO_4_, 2 mM MgCl_2_, 0.2% *w*/*v* defatted BSA). The TC plate containing mitochondria and respiration buffer was incubated at 37 °C for 10 min, then loaded into the Seahorse Analyzer for analysis.

Mitochondria were first treated with 1 mM G3P or Pro to assess the oxygen consumption rate (OCR) under state 4 conditions (substrate alone). After 3-min, ADP was injected into each well at a final concentration of 0.5 mM to interrogate state 3 (phosphorylating) respiration. OCR was read until the ADP was exhausted, which is indicated by a decline in the rate of O_2_ consumption. Mitochondria were then exposed to oligomycin (2.5 µg/µL, an ATP Synthase inhibitor) to assess state 4_O_ (non-phosphorylating; proton leak-dependent) respiration. Note that state 4 and state 4_O_ often yield similar OCR since the concentration of endogenous ADP in mitochondria is low. Finally, antimycin A (4 µM, a Complex III inhibitor) with rotenone (4 µM) and myxthiazol (4 µM) were injected in each well to measure non-mitochondrial O_2_ consumption. All respiration values were corrected for mitochondrial protein equivalents per well and normalized against OCR values from sources independent of the respiratory chain.

### 2.10. Regulation of Complex I Activity and ROS Production

Intact liver mitochondria were diluted to 4 mg/mL and treated with 1000 nM disulfiram and 1 mM GSH as described above. Samples were then washed three times with MESH to remove excess disulfiram and incubated with/without dithiothreitol (DTT; 1 mM) to reduce protein glutathione mixed disulfides. Intact mitochondria were collected, washed thrice, and diluted to 0.5 mg/mL, and the rate of H_2_O_2_ genesis was examined following the addition of G3P and Pro to a final concentration of 1 mM. Rotenone was also included as a control. Rates for ROS production were estimated using AUR.

The activity for complex I was assessed as described in [26]. Briefly, mitochondria from the reactions described above were disrupted by repeated freeze/thaw cycling and diluted to 0.1 mg/mL in MESH-B containing 1 mM KCN, and 100 µM Coenzyme Q. Reactions were supplemented with NADH (100 µM) and its oxidation was tracked for 5 min. Complex I activity was calculated using molar extinction value of ε_340_ = 6220 M^−1^ cm^−1^.

### 2.11. Statistical Analyses and Diagrams

Rates of H_2_O_2_ production were initially calculated in EXCEL using linear standard curves for the reaction of H_2_O_2_ (1–1000 nM) using AUR, HRP, and SOD. Rate results and traces were then collated into Graphpad Prism 9 for statistical analyses. OCR values and enzyme activities were also calculated using EXCEL and Graphpad Prism 9 software. All experiments were conducted four times and in duplicate. Rate results were analyzed by one-way ANOVA with a Fisher’s least significant square post hoc test. * = *p* ≤ 0.05, ** = *p* ≤ 0.01, *** = *p* ≤ 0.005. Diagrams and the Graphical Abstract were generated using Biorender.com.

## 3. Results and Discussion

### 3.1. Disulfiram Augments O_2_^●−^/H_2_O_2_ Production by Liver Mitochondria Oxidizing UQ-Linked Substrates

We had observed previously that glutathionylation catalyst disulfiram increased O_2_^●−^/H_2_O_2_ production when UQ pool substrates G3P, Pro, or succinate were used as fuels for liver mitochondria [25]. We had hypothesized in this previous report that glutathionylation would impede ROS production through inhibition of GPD and PRODH [25]. This hypothesis was formulated based on our previous discovery that glutathionylation of pyruvate dehydrogenase, α-ketoglutarate dehydrogenase, and complex I (when operating in a forward, NADH fueled direction) inhibits mitochondrial O_2_^●−^/H_2_O_2_ genesis (reviewed in [10]). However, in the report using G3P, Pro, and succinate, we found glutathionylation increased ROS production during the oxidation of UQ substrates [25]. Inhibitor studies suggested this increase in ROS production was due to a blockage of electron flow during RET to complex I. In the present study, we set out to examine if complex I glutathionylation accounted for this increase in RET driven ROS genesis. However, before proceeding, we first repeated the experiments published in our recent report on the glutathionylation-mediated increase in ROS production during UQ substrate oxidation [25]. Fueling mitochondria with G3P and Pro confirmed increasing doses of disulfiram augmented ROS production by liver mitochondria (Figure 2). Doses of disulfiram as low as 100 nM induced a significant increase in O_2_^●−^/H_2_O_2_ production when mitochondria were fueled by G3P (Figure 2A). Disulfiram also increased ROS production when Pro was the substrate. Treatment of mitochondria with up to 250 nM disulfiram did not induce any significant changes in O_2_^●−^/H_2_O_2_ production (Figure 2B). However, 500 and 1000 nM disulfiram induced a more than ~2-fold increase in ROS generation (Figure 2B).

**Figure 4:**
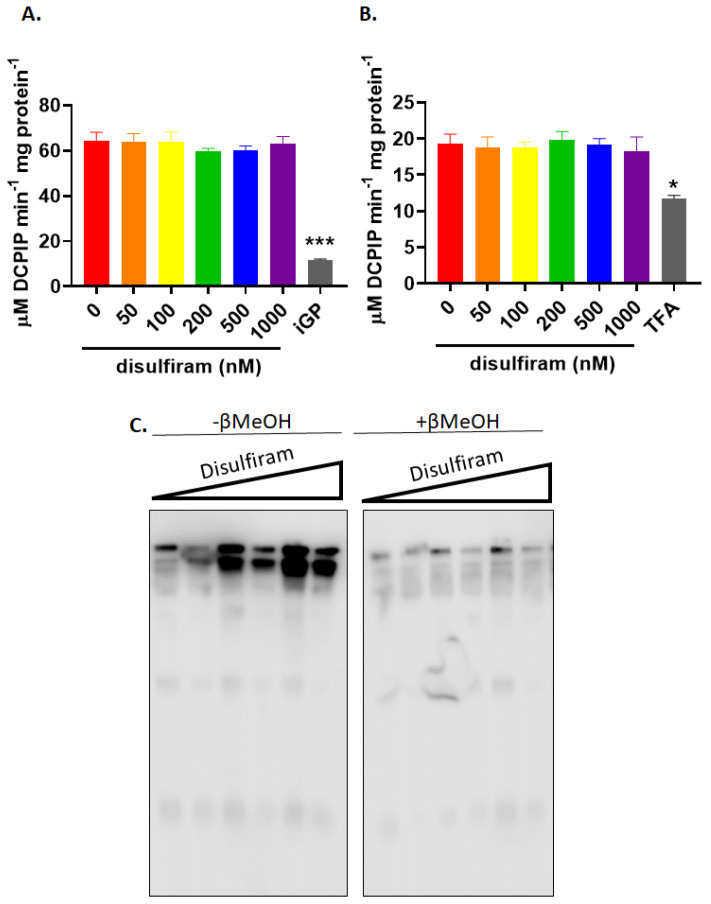
Disulfiram dose dependently increases the total number of protein glutathione mixed disulfides (PSSG) in mitochondria but does not alter the activities of (**A**) GPD and (**B**) PRODH. The activities of both enzymes were estimated using mitochondria treated with increasing doses of disulfiram and 1 mM GSH. The activity of GPD and PRODH was verified with iGP and TFA, respectively. N = 4, mean ± SEM, 1-way ANOVA with a post-hoc Fisher’s LSD test. * *p* ≤ 0.05, *** *p* ≤ 0.001. (**C**) Isolated mitochondria were subjected to immunoblot analysis for PSSG following treatment with disulfiram and 1 mM GSH. Samples were also electrophoresed under reducing conditions (with β-mercaptoethanol; +βMeOH) to confirm the specificity of the protein glutathione disulfide adduct (PSSG) anti-serum.

Next, we examined the sources of ROS in the electron transport chain using a panel of inhibitors for complex I, III, GPD, and PRODH, respectively. GPD and PRODH can generate high amounts of ROS in certain cells, like flight muscles in insects or brown fat [27,28]. However, GPD and PRODH display only modest rates for O_2_^●−^/H_2_O_2_ production in liver, muscle, and cardiac tissue from rodents [27,29,30]. It has also been estimated most of the ROS produced during G3P and Pro oxidation occurs following RET to complex I and FET to complex III [20]. We first interrogated the different sites for this ROS production using complex I and III inhibitors, rotenone and myxothiazol (Figure 3A). Both compounds decrease ROS production by both complexes during RET (complex I; rotenone) and FET (complex III; myxothiazol). Atpenin A5 was included to negate electron transfer to complex II from the UQ pool, eliminating ROS formation by RET to this site (Figure 3A). The rate for ROS production decreased to control levels when mitochondria fueled with G3P or Pro were exposed to either inhibitors (Figure 3B,C). Co-treatment with both rotenone and myxothiazol almost abolished ROS production by mitochondria energized with either substrate (Figure 3B,C). We followed up these experiments using new inhibitors for ROS production by complexes I and III, S1QEL and S3QEL. In contrast to rotenone and myxothiazol, S1QEL and S3QEL reportedly prevent ROS production by complexes I and III without blocking respiration (Figure 3D) [31]. Thus, the advantage to using these two inhibitors is the elimination of interference from artificial electron accumulation in sites for ROS production. We also included experiments with GPD and PRODH inhibitors, iGP and TFA (Figure 3D). Inclusion of S1QEL and S3QEL in the reaction mixtures lowered ROS production in disulfiram-treated mitochondria energized with G3P or Pro (Figure 3E,F). Inclusion of iGP also significantly decreased O_2_^●−^/H_2_O_2_ production in mitochondria fueled with G3P (Figure 3E). We conducted similar experiments in Figure 3F with PRODH inhibitor, TFA. Incubations in 1 mM TFA did not induce a significant decrease in production, even though a trend for a decrease was observed (Figure 3F). Taken together, our observations show UQ-linked substrates can fuel high rates for O_2_^●−^/H_2_O_2_ production by RET to complex I and FET to complex III.

### 3.2. GPD and PRODH Are not Glutathionylation Targets

So far, the results presented above demonstrate glutathionylation can augment ROS production by the electron transport chain when UQ-linked substrates G3P and Pro serve as substrates for mitochondria. iGP and, to a lesser extent, TFA, inhibitors for GPD and PRODH, respectively, also decreased glutathionylation-induced ROS production. This prompted us to investigate the impact of glutathionylation on the activity of both flavoproteins and determine if both GPD and PRODH are targeted for modification with GSH. Figure 4A shows that exposing mitochondria to disulfiram concentrations as high as 1000 nM did not significantly alter the activity of GPD. The activity of GPD under the assay conditions was confirmed using 10 µM iGP, a selective and membrane permeable inhibitor for GPD (Figure 4A). Next, we conducted the same assay except for PRODH. Like GPD, PRODH activity was not affected by disulfiram, even when subjected to doses as high as 1000 nM (Figure 4B). PRODH activity was verified using TFA (Figure 4B). To our knowledge there are few commercially available competitive inhibitors for PRODH. TFA did induce a significant decrease in PRODH activity (Figure 4B). However, even though TFA is structurally analogous to proline, the impact of such a high concentration on PRODH activity was marginal. We followed up these findings by verifying disulfiram was inducing a dose-dependent increase in total number of glutathionylation adducts. As noted in Figure 4C, there is a dose-dependent increase in the band intensity and number of glutathionyl protein adducts in mitochondria treated with disulfiram. Mitochondria treated with reducing agents confirmed the specificity of the protein glutathione disulfide (PSSG) anti-serum (Figure 4C).

**Figure 5:**
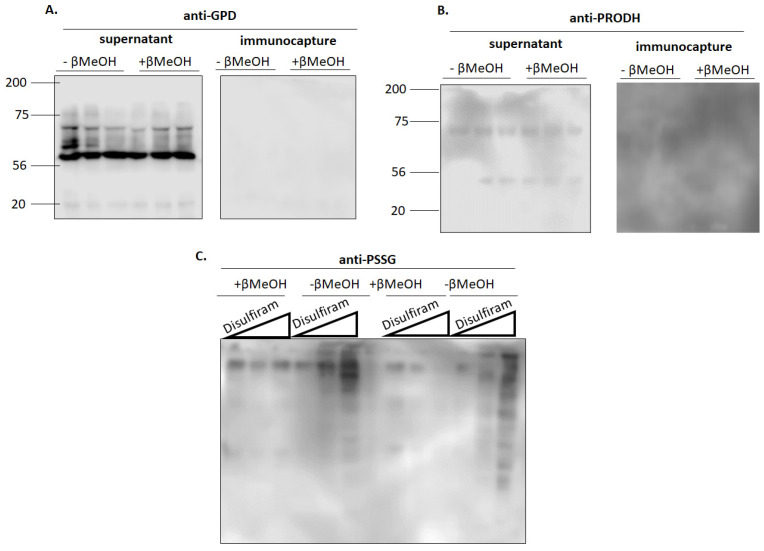
Induction of protein S-glutathionylation does not result in the modification of (**A**) GPD or (**B**) PRODH with glutathione. Estimation of the glutathionylation status of GPD and PRODH was conducted using disulfiram and the BioGEE switch assay. BioGEE modified proteins were isolated using magnetic streptavidin-coated beads and a magnetic rack as described in the Experimental section. The supernatant and the immunocapture fractions were collected and stored at −80 °C for Western blot analysis. Samples treated with β-mercaptoethanol served as a control to verify antibody specificity. N = 3. (**C**) Immunoblot analysis of PSSG adducts after incubation of mitochondria in disulfiram (0, 1000, 10000 nM) and BioGEE (1 mM). Samples also included β-mercaptoethanol (+β-MeOH) to confirm the specificity of PSSG antiserum. N = 2.

The results above demonstrate disulfiram is an effective chemical glutathionylation catalyst that increases mitochondrial ROS production by RET and FET to complexes I and III. However, disulfiram did not affect the activity of GPD and PRODH, suggesting both enzymes are not targeted for modification and that the increase in ROS production is not due to the glutathionylation and inhibition of either flavoprotein. Next, we interrogated the glutathionylation state of GPD and PRODH using the BioGEE switch assay. As noted in the Experimental section, mitochondria were isolated in buffers containing NEM to block and protect unmodified thiols from spontaneous oxidation. Samples were then treated with DTT, desalted, and incubated in NEM free buffer containing 1000 nM disulfiram + 1 mM BioGEE. Proteins modified by BioGEE were isolated using magnetic streptavidin beads. Both the eluant and supernatant collected after the immunocapture were then stored at −80 degrees until analysis. Assessment of samples treated with or without β-mercaptoethanol revealed the presence of immunoreactive bands corresponding to GPD and PRODH only in the supernatant and not in the samples collected from the streptavidin immunocapture (Figure 5A,B). We confirmed the effectiveness of disulfiram in catalyzing the addition of BioGEE to proteins by interrogating the total number of modified targets in mitochondria using PSSG antiserum. Analysis was conducted on mitochondria treated with low (1000 nM) or high (10,000 nM) disulfiram plus BioGEE (Figure 5C). β-mercaptoethanol was included to confirm the specificity of the PSSG antiserum towards PSSG adducts. Disulfiram induced an increase in the total number of glutathionyl-protein adducts in two separate samples (Figure 5C). Inclusion of β-mercaptoethanol confirmed these immunoreactive bands were due to disulfide bridge formation between a proteinaceous thiol and BioGEE. Together, these findings demonstrate the increase in ROS production following the chemical induction of glutathionylation is not due to the modification of GPD or PRODH with GSH.

**Figure 6:**
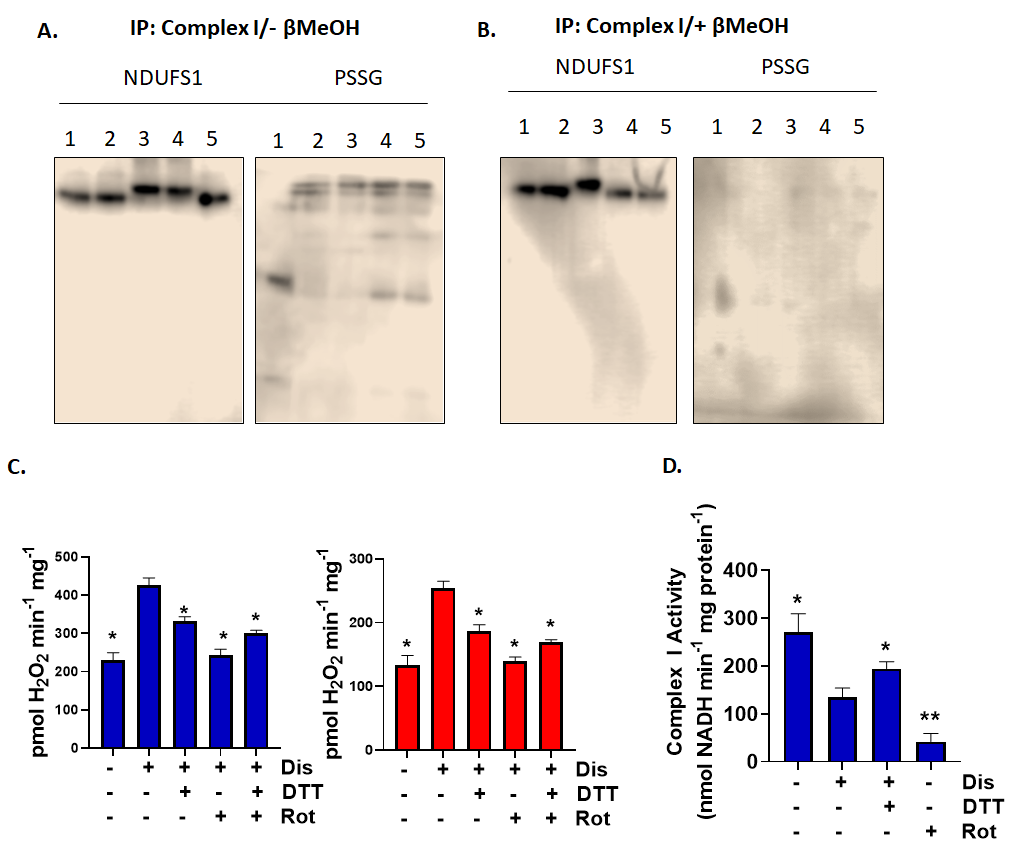
Modification of NDUFS1 in complex I accounts for the increased production of ROS in liver mitochondria oxidizing G3P and Pro. (**A**) Diamide and disulfiram treatment results in the glutathionylation of NDUFS1 subunit of complex I. Mitochondria were exposed to (1) control conditions (no glutathionylation catalyst), (2) 1000 µM diamide, (3) 5000 µM diamide, (4) 5000 nM disulfiram, and (5) 10,000 nM disulfiram and then complex I was immunoprecipitated according to the manufacturer’s instructions. (**B**) Mitochondria treated with β-mercaptoethanol served as a control to verify PSSG anti-serum specificity. NDUFS1 was also detected to demonstrate it was successfully immunocaptured. (**C**) Reversal of glutathionylation diminishes ROS production in mitochondria oxidizing G3P or Pro. Mitochondria were treated with 1000 nM disulfiram + 1 mM GSH, washed several times, and incubated with DTT. Rotenone was also added to some reactions as a control. N = 4, mean ± SEM, 1-way ANOVA with a post-hoc Fisher’s LSD test. * *p* ≤ 0.05. (**D**) DTT treatment restores the activity of complex I after treatment with disulfiram. Mitochondria were incubated in disulfiram + GSH as described in C. and then DTT was added. N = 4, mean ± SEM, 1-way ANOVA with a post-hoc Fisher’s LSD test. * *p* ≤ 0.05, ** *p* ≤ 0.01.

### 3.3. Blocking Electron Flow through Glutathionylation of Complex I Subunit NDUFS1 Increases RET-Driven ROS Production during G3P and Pro Metabolism

Complex I contains 46 subunits in total and several are targeted for glutathionylation [5]. However, to date, only the glutathionylation of NDUFS1 and NDUFV1 has been found to affect the activity of complex I [11]. Most work has focused on NDUFS1 due to the relationship between its modification with glutathione and the impact it has on complex I activity and ROS production in several pathologies [23,32]. The findings in this report, so far, indicate disulfiram treatment augments RET-driven O_2_^●−^/H_2_O_2_ production by complex I. Blockage of electron flow to NAD^+^ during RET due to NDUFS1 glutathionylation may result in the over-reduction of FMN or the UQ site (both are known sites for production in complex I) resulting in a higher rate of ROS production. Therefore, we conducted an immunocapture assay to examine the glutathionylation state of complex I subunit, NDUFS1. Samples were treated with either disulfiram or diamide, a second catalyst for the chemical induction of glutathionylation. Complex I was immunocaptured and samples were probed with NDUFS1 and protein glutathione mixed disulfide (PSSG; glutathionylation) anti-serum. Samples were also treated with or without β-mercaptoethanol (βMeOH) to confirm the specificity of the PSSG antiserum towards glutathionylated proteins. Immunoblot analyses revealed that we successfully isolated complex I, which was confirmed using anti-NDUSF1 (Figure 6A,B). Slight shifts in mobility were observed, which we attributed to an artifact associated with the use of this immunocapture kit [12]. Interrogating blots with PSSG antiserum also revealed the presence of several immunoreactive bands. The high intensity bands were found to correspond with the mobility of NDUFS1 and were only present in mitochondrial samples treated with glutathionylation catalysts (Figure 6A). Inclusion of β-mercaptoethanol resulted in the reduction of the protein-glutathionyl disulfide bridge, abolishing most immunoreactive bands corresponding to PSSG, confirming NDUFS1 is glutathionylated by disulfiram and diamde (Figure 6B).

We followed up these findings by performing assays to evaluate the impact of glutathionylation on ROS production by complex I in liver mitochondria fueled with G3P or Pro. To do so, mitochondria were treated with disulfiram to induce glutathionylation and then treated with dithiothreitol (DTT) to reverse the modification. Incubation of mitochondria in rotenone served as a control. Disulfiram increased ROS production in liver mitochondria oxidizing either substrate (Figure 6C). Incubation of samples in DTT induced a significant decrease in ROS production, indicating blockage of electron flow by reverse transfer from the UQ pool by glutathionylation accounts for the increase in ROS production (Figure 6C). Rotenone alone did decrease the rate of production back to control levels (Figure 6C). Surprisingly, inclusion of rotenone in DTT-treated samples did not lower ROS further (Figure 6C). It was previously shown that DTT can autocatalyze the conversion of Amplex UltraRed to fluorescent resorufin [33]. We avoided desalting samples, so mitochondria remained intact for our analyses. To do this, we opted to wash our reactions several times prior to conducting assays. However, it is likely small amounts of contaminating DTT remained even after the washes. Therefore, despite several washes prior to conducting the O_2_^●−^/H_2_O_2_ measurements, contaminating DTT may have interfered with the assay by autocatalyzing resorufin production. However, DTT has also been shown to reactivate complex I and recover mitochondrial ATP production following its inhibition by glutathionylation [13,23]. Therefore, we also examined complex I activity in response to disulfiram-induced glutathionylation and its reversal with DTT. Induction of the glutathionylation inhibited the activity of complex I, which could be partially reversed by a DTT incubation (Figure 6D).

Complex I of the respiratory chain has multiple glutathionylation sites, including NDUFS1, NDUFV1, and NDUFA11 [22]. Proteomic analyses of exercised rat muscle tissue also identified several other complex I subunits that can be modified by glutathionylation [5]. To date, only the glutathionylation of NDUFS1 and NDUFV1 have been found to have a functional effect on complex I and NADH-driven respiration [11,13]. Furthermore, most work has focused on NDUFS1because it was the first glutathionylation site identified in mitochondria and a target for reversible glutathionylation in response to GSH pool oxidation state. GRX2 drives glutathionylation of complex I in response to GSH pool oxidation which inhibits its activity and deactivates NADH-induced O_2_^●−^/H_2_O_2_ production [11,12]. Restoration of the redox state of the GSH pool by NADPH drives the GRX2-mediated deglutathionylation of complex I, restoring its activity and NADH-dependent respiration. The temporary S-glutathionylation of NDUFS1 has been hypothesized in several articles to serve as a mechanism to prevent oxidative distress and damage by protecting vulnerable thiols from over-oxidation while simultaneously limiting ROS production for antioxidant defense system recovery [12,13]. For example, modification of Cys-531 and Cys-704 on NDUFS1 protects the subunit from irreversible oxidation when H_2_O_2_ levels are high or during nitrosative stress [13]. Removal of GSH from NDUFS1 restores the activity of the enzyme. Additionally, the temporary deactivation of complex I limits ROS production by the respiratory complex and electron transport chain during NADH oxidation, preventing antioxidant defense depletion and oxidative damage [12]. Similar observations have been made with α-ketoglutarate dehydrogenase and pyruvate dehydrogenase [26,34]. Protein S-glutathionylation limits ROS production by α-keto acid dehydrogenases, which also has the added benefit of protecting the thiols in the lipoamide of the E2 subunit from over-oxidation [26,34,35]. Thus, the reversible modification of mitochondrial ROS generators like the α-keto acid dehydrogenases and complex I plays an integral role in preventing oxidative distress. Moreover, this mechanism is likely required for controlling H_2_O_2_ availability in mitochondria-to-cell signaling [10]. Overall, the GRX2-mediated reversible modification of complex I subunit, NDUFS1, and other ROS sources is integral for maintaining mitochondrial and cellular redox balance for cell signaling and the prevention of oxidative distress.

In 2003, Taylor et al. demonstrated glutathionylation increased O_2_^●−^/H_2_O_2_ production by complex I in bovine heart mitochondria subjected to oxidative distress [21]. This occurred through the formation of PSSG adducts on 51 and 75 KDa subunits, both of which were identified later as NDUFV1 and NDUFS1, respectively [21]. NDUFS1 was also identified as a major site for the spontaneous and prolonged modification of complex I by glutathionylation in several pathologies including the development of cataracts, sarcopenia, and heart disease [23,32,36]. This was related to the depletion of GSH pools, over production of mitochondrial ROS, cell damage, and oxidative distress, resulting in dysfunctional oxidative phosphorylation and nutrient metabolism. Recent work has shown that mitochondrial respiration can be restored by reversing complex I glutathionylation and preserving the reductive potential of the GSH pool, which can have a protective effect against metabolic and cardiovascular diseases [36,37,38]. However, the source of ROS following prolonged complex I deactivation by glutathionylation due to GSH pool oxidation has remained elusive. Here, we have demonstrated for the first time that glutathoinylation-mediated blockage of electron flow through complex I during RET can increase ROS production. This is due, in part, to the modification of NDUFS1, a critical subunit required for forward and reverse electron transfer reactions in complex I. These findings are highlighted by the reversibility of the modification of NDUFS1, which was induced by the reducing agents. Furthermore, removal of the glutathionyl moiety with DTT restored the activity of complex I and partially protected from increased RET-induced ROS production. Collectively, these findings provide evidence that glutathionylation of NDUFS1 can result in an increase in ROS production when nutrients that by-pass complex I and donate electrons directly to the UQ pool are being oxidized by mitochondria.

### 3.4. Disulfiram Treatment Inhibits G3P and Pro Fueled ATP Production

**Figure 7:**
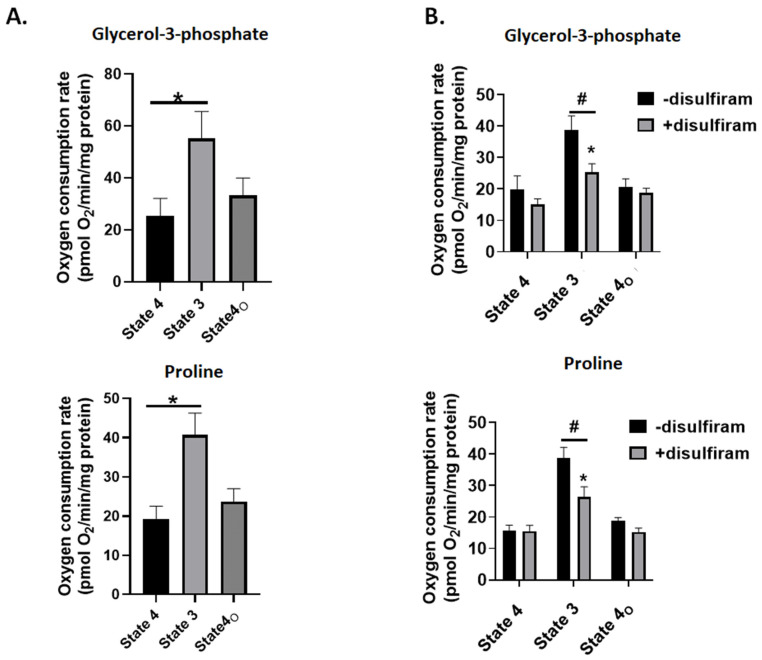
Treatment of mitochondria with disulfiram inhibits state 3 respiration, indicating it may inhibit complex III. (**A**) Mitochondria energized with glycerol-3-phosphate or proline exhibit measurable rates of state 4 (substrate alone), state 3 (injection of ADP), and state 4_O_ (treatment with complex V inhibitor, oligomycin). State 4 and state 4_O_ represent measures of non-phosphorylating or proton leak-dependent respiration. *n* = 4, mean ± SEM, 1-way ANOVA with a post hoc Fisher’s LSD test. * *p* ≤ 0.05. (**B**) Mitochondria were seeded in Seahorse TC plates and treated with 1000 nM disulfiram and 1 mM GSH. Assays were then conducted as described in (**A**) and the Experimental section. *n* = 4, mean ± SEM, 2-way ANOVA with a post hoc Fisher’s LSD test. * *p* ≤ 0.05 when conducting statistical comparisons within a group, # *p* ≤ 0.05 when conducting comparisons between control and disulfiram-treated groups.

Myxothiazol and S3QEL also lowered disulfiram-induced ROS production during G3P and Pro. Reduction of the UQ pool by GPD and PRODH can also stimulate ROS production by RET to complex II [27,39]. However, atpenin A5, which inhibits ROS production by complex II during RET by blocking the UQ binding site, was also included in our experiments. Taken together, the results in Figure 3 indicate complex III is a potential ROS source following disulfiram-induced glutathionylation, which may be related to the blockage of the chain upstream from complex III. To determine if the ETC was blocked, we assessed the different states of respiration using liver mitochondria oxidizing either G3P or Pro. For this, we used a Seahorse XFe24 method, which relies on attaching mitochondria to the surface of an XFe24 tissue culture plate. This method has been applied to study the impact of reversible S-glutathionylation and the loss of GRX2 on mitochondrial bioenergetics, specifically the capacity to oxidize complex I-linked substrates such as pyruvate or glutamate [23]. However, to our knowledge, this method has not been used to study the oxidation of UQ linked substrates such as G3P and Pro. As a proof of concept, we first assessed the different states of respiration in liver mitochondria oxidizing G3P or Pro. Figure 7A shows injection of ADP into individual wells containing mitochondria increased the rate of O_2_ consumption (state 3). Additionally, phosphorylating respiration was successfully blocked by injecting oligomycin into each well to induce proton leak-dependent respiration (state 4_O_) (Figure 7A). Taken together, G3P and Pro-fueled O_2_ metabolism can be measured in the Seahorse XFe24.

Next, we tested if glutathionylation impeded mitochondrial O_2_ metabolism during G3P and Pro oxidation. Treatment of liver mitochondria oxidizing either substrate with 1000 nM disulfiram did not significantly change state 4 (substrate alone) or state 4_O_ respiration (treatment with oligomycin) (Figure 7B). Measurement of state 3 respiration revealed that disulfiram treatment induced a significant decrease in the rate of O_2_ consumption in mitochondria oxidizing either G3P or Pro (Figure 7B). Complex III is the major oxidant source in mammalian cells and supplies H_2_O_2_ to activate a myriad of signaling pathways. Complex III has been identified as a glutathionylation target using redoxomics and clickable glutathione analogs in cardiomyocytes [40]. The findings presented in Figure 3 and Figure 7B, as well as in [25], suggest that induction of glutathionylation also increases ROS production by complex III when UQ substrates serve as fuel. However, it should be noted we did not generate any evidence this increase is due to complex III glutathionylation. Indeed, the increase could be due to the blockage of electron flow further downstream from complex III. For example, complex V glutathionylation results in lowered respiration and oxidative distress [41]. This could induce an increase in H_2_O_2_ generation since complex V inhibitors like oligomycinaugment mitochondrial oxidant production due to blockage of the proton circuit. Complex IV is also a potential glutathionylation site, which would also increase ROS genesis by mitigating electron flow to the O_2_ binding site [42]. In aggregate, the induction of glutathionylation does increase ROS production by complex III following oxidation of UQ-linked substrates. Whether this is due to complex III modification or glutathionylation of complexes upstream from the UQ-cytochrome C oxidoreductase needs to be elucidated.

## 4. Conclusions

Mitochondria are critical sources of cellular H_2_O_2_, a second messenger utilized to modify cell behaviour in response to extracellular stimuli and changing energy demands. These signals are mediated through the oxidation and reduction of cellular protein cysteine thiols called “redox relays”. Importantly, the use of H_2_O_2_ as a “mitokine” relies on having tight control over its production, subsequent availability, and degradation. There are 16 sources of mitochondrial H_2_O_2_, 12 of which are associated with nutrient metabolism and oxidative phosphorylation. Complexes I and III have been identified as major redox signaling platforms, with little attention being given to the other 10 mitochondrial ROS sources. This is surprising given that flavoenzymes such as the α-ketoglutarate and pyruvate dehydrogenases have been shown to display high rates of ROS production and are major mitochondrial redox sensors [20]. GPD and PRODH also have measurable rates of ROS production and can generate significant amounts by electron transfer to the respiratory chain. Whether these generators, or other sources like GPD or PRODH, contribute to cell signaling remains to be determined.

Control over H_2_O_2_ availability is required to elicit rapid responses in cell behaviour but is also vital for preventing oxidative distress. Protein S-glutathionylation has emerged as an integral mechanism for the feedback inhibition of mitochondrial ROS production. Oxidation of the GSH pool results in the modification of complex I and other flavin dependent enzymes, which inhibits ROS production and desensitizes H_2_O_2_ signals. This also allows for the NADPH-dependent restoration of the reductive capacity of the GSH pool and other antioxidants, culminating with the deglutathionylation of these ROS producers and the reactivation of enzyme activities and mitochondrial nutrient metabolism. However, oxidative distress, cell damage, and the prolonged oxidation of GSH pools can have the opposite effect, resulting in the over or hyper glutathionylation of mitochondrial proteins and a subsequent increase in ROS production, and inhibition of ATP generation. Here, we successfully demonstrated for the first time the chemical induction of glutathionylation augments ROS production by RET to complex I and FET to complex III. We show this is related to blockage of RET from the UQ pool through complex I, specifically through the modification of NDUFS1 with glutathione. This observation accounts for the increased ROS production observed in pathological conditions associated with the over or prolonged glutathionylation of complex I and the mitochondrial proteome during oxidative distress. We also generated evidence showing modification of respiratory complexes or other important elements of the respiratory chain (e.g., ATP synthase) with glutathione can also enhance ROS production during FET from the UQ pool. This increase in ROS production during FET is likely associated with the over-reduction of complex III due to decreased electron flow through the chain. Overall, our findings provide important insights into the sources of augmented ROS production during oxidative distress, which has strong implications for understanding how dysfunctional glutathionylation reactions may contribute to disease progression.

## Data Availability

Data are available upon request from the corresponding author.
